# Supplementary material for: Dynamic CpG methylation delineates subregions within super-enhancers selectively decommissioned at the exit from naive pluripotency
Source: Nat Commun. 2020 Feb 28;11:1112. doi: 10.1038/s41467-020-14916-7 (PMC7048827; doi:10.1038/s41467-020-14916-7)
Supplement: Supplementary file 1 — Supplementary Information [file 41467_2020_14916_MOESM1_ESM.pdf]

Dynamic CpG Methylation Delineates Subregions within Super-Enhancers Selectively Decommissioned at the Exit from Naïve Pluripotency

Bell *et al.*

Supplementary Data 1: List of all datasets used with accession numbers

Supplementary Data 2: Capture Hi-C data (Joshi *et al.* and Sahlen *et al*.)

Supplementary Data 3: CpG methylation and ATAC-seq at SE subregions

Supplementary Data 4: ROC analysis in naïve-like and primed-like single ESC clusters

Supplementary Data 5: Motif enrichment and expression of corresponding transcription factors (TFs) at SE subregions

Supplementary Data 6: List of qPCR primers used for expression analysis as well as 5mC and ChIP assays

Supplementary Data 7: 4C-seq primers and mapping statistics

Supplementary Data 8: Coefficients and statistics for logistic regression models relating epigenetic features to SE subregion status

Supplementary Data 9: Coefficients and statistics for multivariable linear regression model relating ESRRB and OCT4 DNA-binding at PU vs DM to the loss of MED1 binding upon constitutive depletion of ESRRB

Supplementary Data 10: Source Data file for all relevant Main Figures and Supplementary Figures

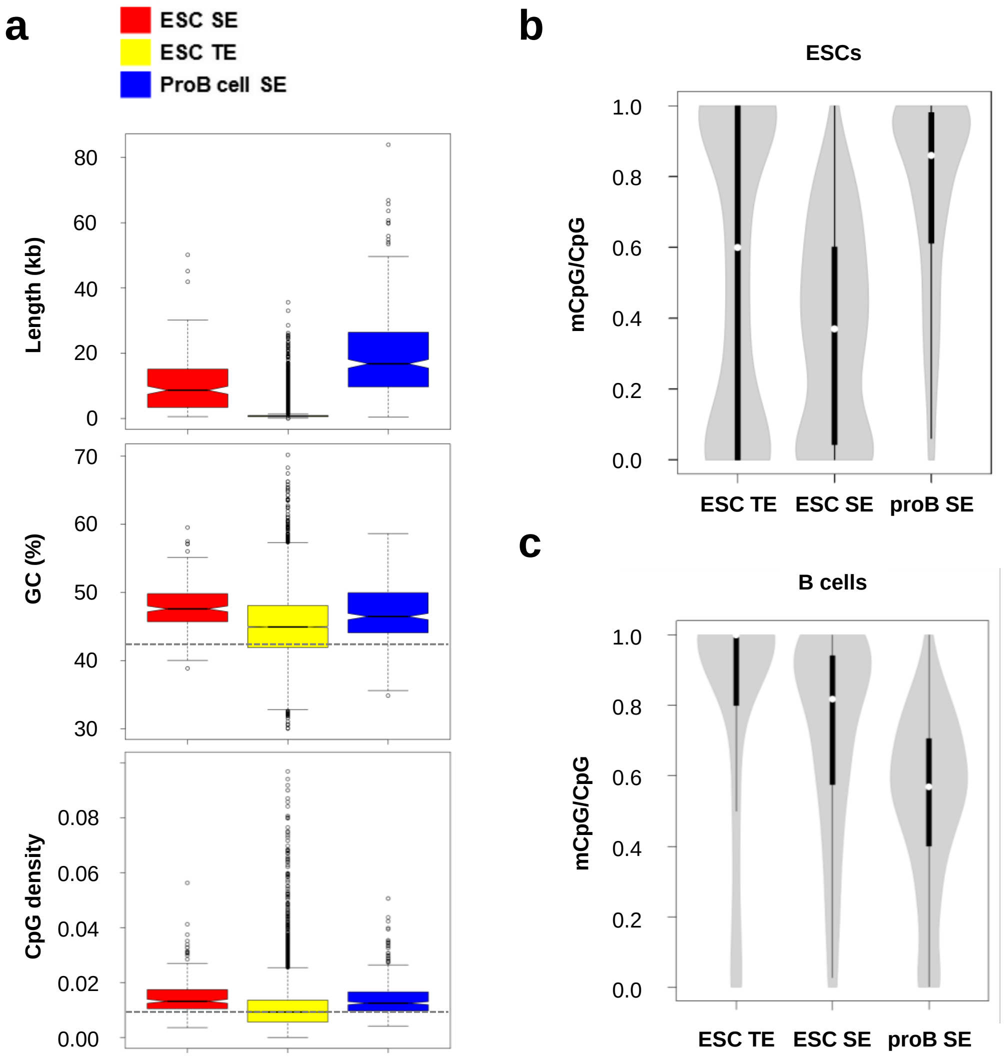

Supplementary Figure 1. **Related to Figure 1 - Comparative analysis of typical enhancers and super-enhancers mapped in ESCs and somatic cells**

(**a**) Features of embryonic stem cell (ESC) typical-enhancers (TEs; yellow), ESC super-enhancers (SEs; red) and proB cell (blue) SEs: length in kb, % of GC and CpG density calculated as the number of CpG dinucleotides within a region divided by its length. Dotted lines on the second and third plots indicate global averages in the mouse genome. (**b, c**) CpG methylation levels (mCpG/CpG) in ESCs grown under serum/LIF conditions[1] (**b**) or B cells[2] (**c**) at ESC-specific TEs, ESC-specific SEs and proB cell-specific SEs.

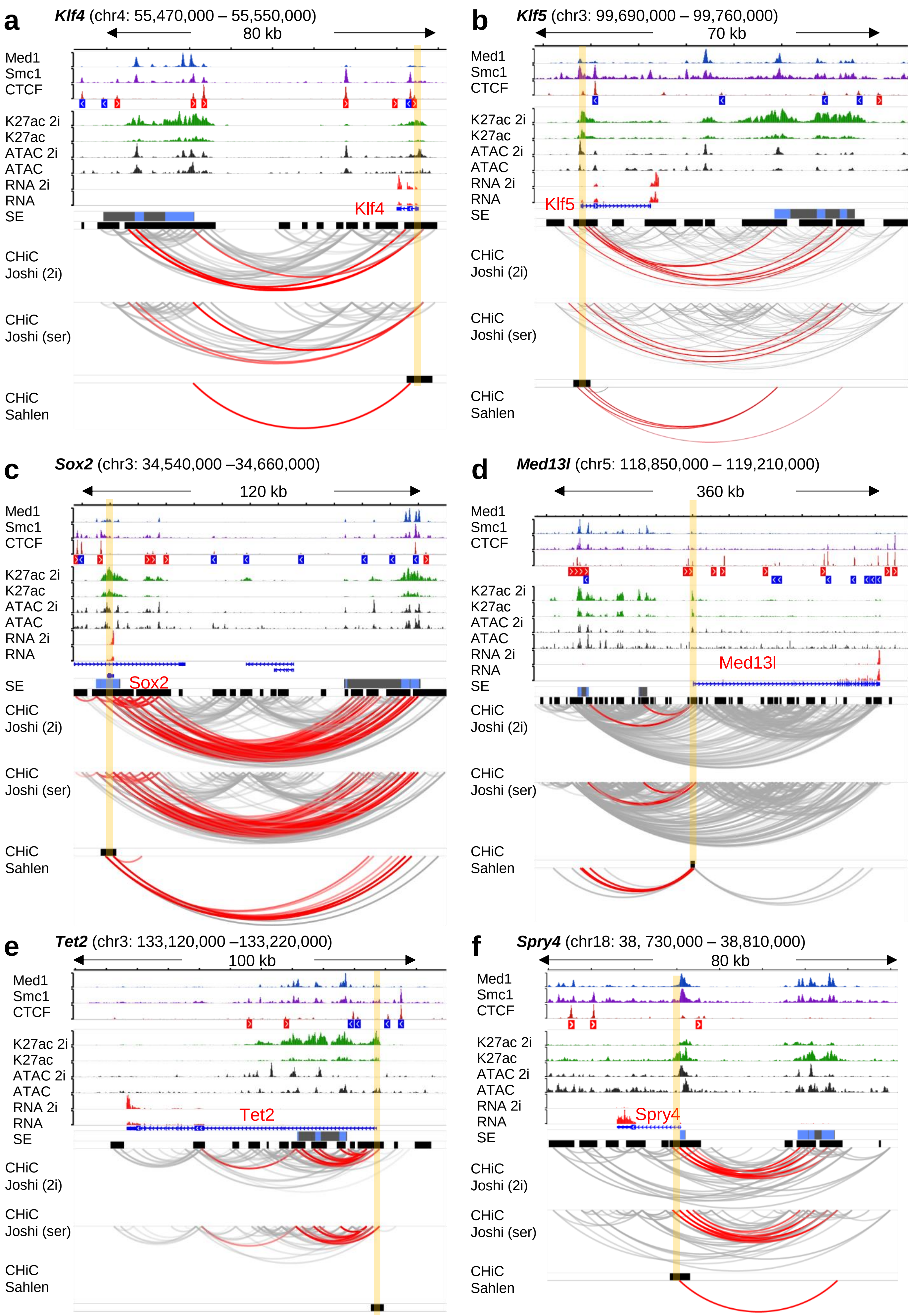
a
Klf4 (chr4: 55,470,000 – 55,550,000)
80 kb
Med1
Smc1
CTCF
K27ac 2i
K27ac
ATAC 2i
ATAC
RNA 2i
RNA
SE
Klf4
CHiC Joshi (2i)
CHiC Joshi (ser)
CHiC Sahlen
b
Klf5 (chr3: 99,690,000 – 99,760,000)
70 kb
Med1
Smc1
CTCF
K27ac 2i
K27ac
ATAC 2i
ATAC
RNA 2i
RNA
SE
Klf5
CHiC Joshi (2i)
CHiC Joshi (ser)
CHiC Sahlen
c
Sox2 (chr3: 34,540,000 –34,660,000)
120 kb
Med1
Smc1
CTCF
K27ac 2i
K27ac
ATAC 2i
ATAC
RNA 2i
RNA
SE
Sox2
CHiC Joshi (2i)
CHiC Joshi (ser)
CHiC Sahlen
d
Med13l (chr5: 118,850,000 – 119,210,000)
360 kb
Med1
Smc1
CTCF
K27ac 2i
K27ac
ATAC 2i
ATAC
RNA 2i
RNA
SE
Med13l
CHiC Joshi (2i)
CHiC Joshi (ser)
CHiC Sahlen
e
Tet2 (chr3: 133,120,000 –133,220,000)
100 kb
Med1
Smc1
CTCF
K27ac 2i
K27ac
ATAC 2i
ATAC
RNA 2i
RNA
SE
Tet2
CHiC Joshi (2i)
CHiC Joshi (ser)
CHiC Sahlen
f
Spry4 (chr18: 38, 730,000 – 38,810,000)
80 kb
Med1
Smc1
CTCF
K27ac 2i
K27ac
ATAC 2i
ATAC
RNA 2i
RNA
SE
Spry4
CHiC Joshi (2i)
CHiC Joshi (ser)
CHiC Sahlen

Supplementary Figure 2. Related to Figure 1 - Promoter-SE interactions detected by capture Hi-C in six example loci in ESCs

The direction of the CTCF motif is denoted below the CTCF track. Unmethylated (blue) and methylated (grey) regions of the super-enhancers (SEs) are shown. DpnII restriction fragments are shown on top of the capture Hi-C tracks. Capture baits are shown as black boxes. Target promoters are marked by yellow band. Promoter-SE and SE-SE interactions are red, other interactions are grey. (**a**) The *Klf4* promoter interacts mostly with the accessible sites of its upstream SE. These accessible sites are unmethylated. (**b**) The *Klf5* promoter interacts mainly with the accessible (unmethylated) regions of the downstream SE. (**c**) *Sox2* locus. Strong interactions between the *Sox2* promoter and the proximal and distal SE. The two SEs also interact with each other and form an insulated neighbourhood bounded by multiple CTCF sites with converging motif orientation. (**d**) *Med13l* and two SEs also appear to be trapped in an insulated neighbourhood in which the SEs, the TSS and the TES are in close proximity. (**e**) The *Tet2* promoter is close to the SE and no significant interaction was found in the Sahlen *et al*. dataset. (**f**) Significant interactions between the *Spry4* promoter and its SE were also found in both Joshi *et al*.[3] and Sahlen *et al*.[4] dataset.

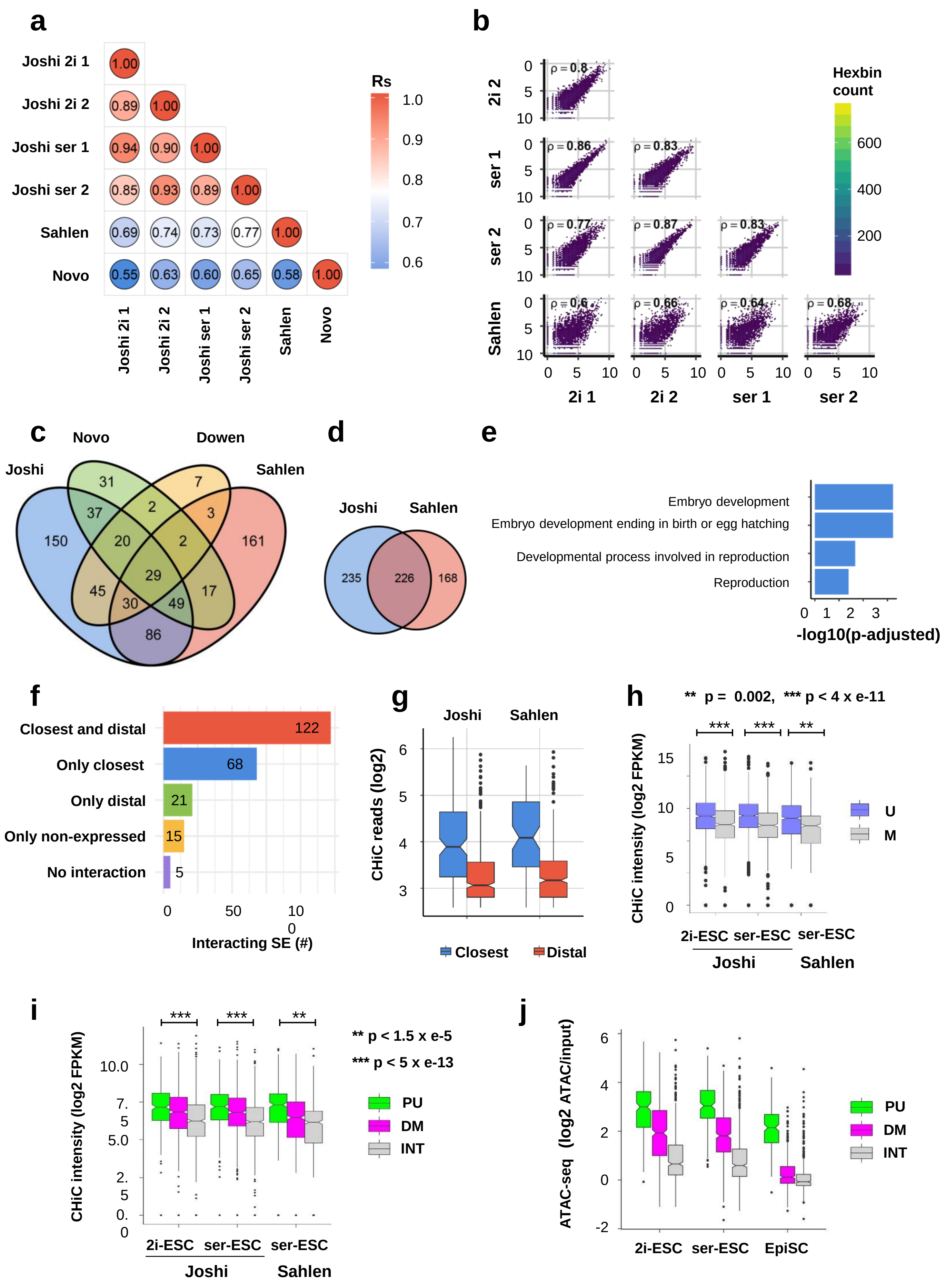

a
Joshi 2i 1
Joshi 2i 2
Joshi ser 1
Joshi ser 2
Sahlen
Novo
1.00
0.89 1.00
0.94 0.90 1.00
0.85 0.93 0.89 1.00
0.69 0.74 0.73 0.77 1.00
0.55 0.63 0.60 0.65 0.58 1.00
Rs
1.0
0.9
0.8
0.7
0.6
b
2i 2
ser 1
ser 2
Sahlen
ρ = 0.8
ρ = 0.86
ρ = 0.83
ρ = 0.77
ρ = 0.87
ρ = 0.83
ρ = 0.6
ρ = 0.66
ρ = 0.64
ρ = 0.68
2i 1
2i 2
ser 1
ser 2
Hexbin count
600
400
200
c
Novo
Dowen
Joshi
Sahlen
31
7
37
2
3
150
20
2
161
29
45
17
30
49
86
d
Joshi
Sahlen
235
226
168
e
Embryo development
Embryo development ending in birth or egg hatching
Developmental process involved in reproduction
Reproduction
-log10(p-adjusted)
f
Closest and distal
Only closest
Only distal
Only non-expressed
No interaction
122
68
21
15
5
Interacting SE (#)
g
Joshi
Sahlen
CHiC reads (log2)
Closest
Distal
h
** p = 0.002, *** p < 4 x e-11
CHiC intensity (log2 FPKM)
U
M
2i-ESC
ser-ESC
ser-ESC
Joshi
Sahlen
i
** p < 1.5 x e-5
*** p < 5 x e-13
CHiC intensity (log2 FPKM)
PU
DM
INT
2i-ESC
ser-ESC
ser-ESC
Joshi
Sahlen
j
ATAC-seq (log2 ATAC/input)
PU
DM
INT
2i-ESC
ser-ESC
EpiSC

Supplementary Figure 3. Related to Figure 1 and Figure 3 - Comparative analysis of capture Hi-C interaction datasets collected from ESCs

(**a**) Spearman rank correlation between the promoter-SE interaction intensity (log2 normalized reads) in different datasets (Joshi *et al*.[3], Sahlen *et al*.[4], Novo *et al*.[5]). For each pairwise comparison, only the promoter-SE interactions for which the promoter contained a capture bait in both datasets were considered. (**b**) Scatterplots of the significant capture Hi-C interaction intensities at promoter-SE subregions. The higher resolution (promoter-SE subregions) compared to promoter-SE in (**a**) results in a lower Spearman correlation between the libraries. The HindIII fragments (Novo *et al.* dataset) often cannot be assigned to a single SE subregion and were omitted for the SE subregion interaction study. (**c**) Significant promoter-SE interactions detected in the three capture Hi-C datasets and one CHIA-PET dataset (Dowen *et al*.[6]). Most promoter-SE interactions overlap with the Joshi *et al.* and Sahlen *et al*. datasets. (**d**) Overlap between the significant promoter-SE interactions found in Joshi et al. and Sahlen et al. datasets. (**e**) Gene set enrichment analysis of all genes that have a significant promoter-SE interaction. Target genes were ranked by their gene-expression fold change between embryonic stem cells (ESCs) in serum/LIF and epiblast stem cells (EpiSCs). P-values were derived from 1 million random permutations and adjusted for multiple testing (Benjamini Hochberg correction). (**f**) Distribution of significant capture Hi-C interactions per SE (union Joshi and Sahlen). (**g**) Capture Hi-C intensity (log2 normalized reads) between SEs and the proximal (closest)/distal promoter targets. Closest SE target genes are defined as the nearest expressed genes (RPKM ≥ 1) to the SEs (see also Methods). As expected, SE proximal promoters have higher interaction frequencies compared to distal promoters. (**h**) Comparison of Capture Hi-C intensities (log2 FPKM) at unmethylated (U; blue) or methylated (M; grey) regions of SEs in ESCs in 2i/LIF (2i) or serum/LIF (ser) culture conditions. (**i**) Comparison of Capture Hi-C intensities (log2 FPKM) at PU (green), DM (magenta) and INT (grey) subregions in ESCs in 2i/LIF or serum/LIF culture conditions. P-values (ANOVA, controlling for the number of capture Hi-C baits per SE subregion and the log2 promoter – SE subregion distance) are indicated. (**j**) Chromatin accessibility at PU/DM/INT subregions in ESCs in 2i/LIF or serum/LIF culture conditions and EpiSCs.

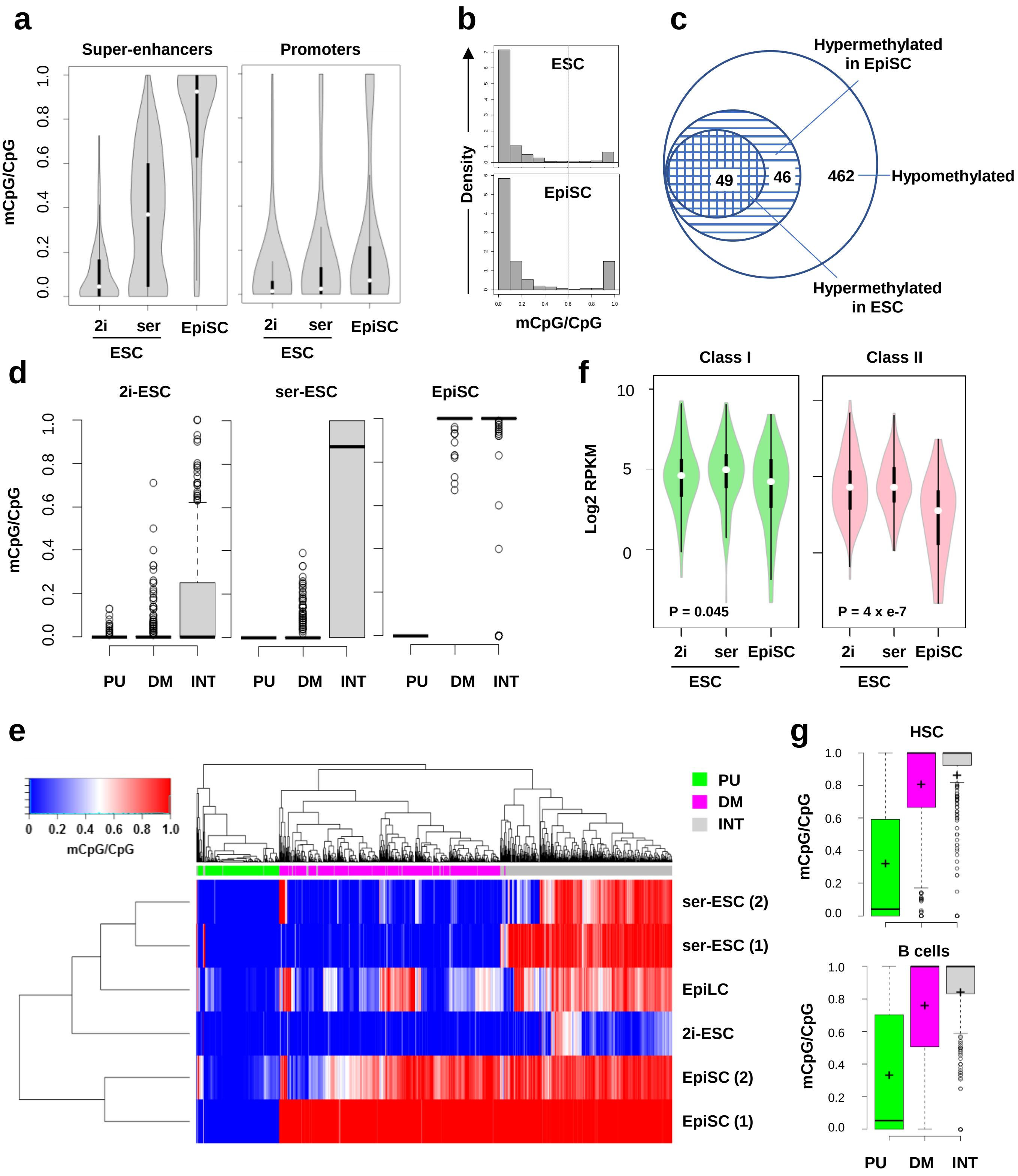

a
Super-enhancers
Promoters
mCpG/CpG
2i ser EpiSC
ESC
b
Density
ESC
EpiSC
mCpG/CpG
c
Hypermethylated in EpiSC
49
46
462
Hypomethylated
Hypermethylated in ESC
d
2i-ESC
ser-ESC
EpiSC
mCpG/CpG
PU DM INT
f
Class I
Class II
Log2 RPKM
P = 0.045
P = 4 x e-7
2i ser EpiSC
ESC
e
mCpG/CpG
PU
DM
INT
ser-ESC (2)
ser-ESC (1)
EpiLC
2i-ESC
EpiSC (2)
EpiSC (1)
g
HSC
B cells
mCpG/CpG
PU DM INT

Supplementary Figure 4. Related to Figure 2 and Figure 3 - CpG methylation distribution at super-enhancers and interacting gene promoters in pluripotent and somatic cells

(**a**) CpG methylation levels (mCpG/CpG) at SEs and promoters in ESCs grown in 2i/LIF (2i)[1] and serum/LIF (ser)[1] and epiblast stem cells (EpiSCs; this study). 2i/LIF conditions known to promote hypomethylation in ESCs serve here as reference. (**b**) Density plots showing the distribution of mCpG/CpG values at SE-associated gene promoters in ser-ESCs and EpiSCs as above. (**c**) Venn Diagram showing the overlap in hypermethylated (ratio mCpG/CpG ≥0.6, dashed line in b) ESC SE-associated gene promoters in ESCs (serum/LIF) and EpiSCs with the majority of ESC SE-interacting promoters remaining hypomethylated in both cell types. (**d**) CpG methylation levels (mCpG/CpG) at PU, DM and interstitial INT subregions in 2i-ESCs and ser-ESCs, and EpiSCs. (**e**) Heatmap showing the proportion of methylated CpGs in PU (green), DM (magenta) and INT (grey) subregions in ESCs, epiblast-like cells (EpiLCs), and EpiSCs. 2i-ESCs, ser-ESCs (1) are from Ref.[1] and EpiSCs (2) from this study as shown in (a-d); ser-ESCs (2) from Ref.[7]; EpiLCs and EpiSCs (2) are from Ref.[8]. Cell-types were clusters by Pearson correlation and PU, DM and INT SE subregions by Euclidian distance. (**f**) Expression (Log2 RPKM) of closest interacting gene promoters associated with Class I (light green) or Class II (pink) SEs in 2i-ESCs and ser-ESCs[9] and in EpiSCs[10]. P-values (Kruskal-Wallis test) are indicated. (**g**) Fate of CpG methylation levels (mCpG/CpG) at PU, DM and INT subregions in haematopoietic stem cells (HSCs)[11] or B cells[2]. See Supplementary Data 1 for dataset accession numbers.

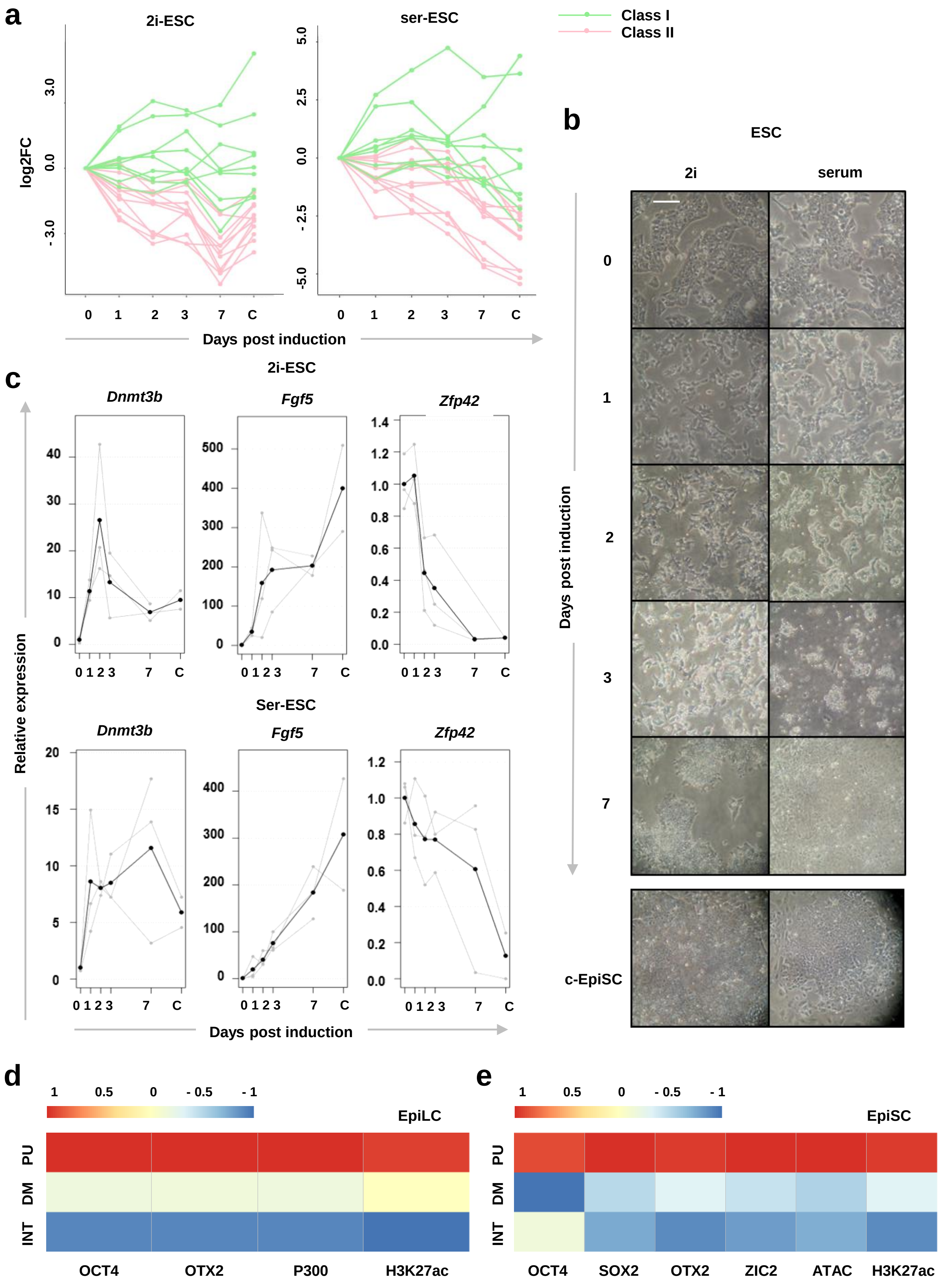

a
2i-ESC
ser-ESC
Class I
Class II
log2FC
3.0
0.0
- 3.0
5.0
2.5
0.0
- 2.5
-5.0
0 1 2 3 7 C
Days post induction
b
ESC
2i
serum
0
1
2
3
7
Days post induction
c-EpiSC
c
2i-ESC
Dnmt3b
Fgf5
Zfp42
Ser-ESC
Relative expression
Days post induction
d
EpiLC
PU
DM
INT
OCT4
OTX2
P300
H3K27ac
e
EpiSC
OCT4
SOX2
OTX2
ZIC2
ATAC
H3K27ac
1 0.5 0 - 0.5 - 1

Supplementary Figure 5. Related to Figure 3 - Activity of PU and DM super-enhancer subregions upon ESC-to-EpiSC conversion and in EpiSCs

(a) Log2(fold change) relative to the starting ESC population of selected genes associated with Class I (light green) or Class II (pink) SEs during conversion from 2i-ESCs (left panel) and ser-ESCs to EpiSCs (right panel) as in Figure 3d. Individual trajectories are shown at different days post induction and in converted EpiSCs (C). Similar results were observed when conversion was induced from 2i-ESCs and ser-ESCs. Data are representative of three independent experiments (n=3). **(b)** Microphotographs of cells during conversion from ESCs to EpiSCs. Scale bar = 200µm. **(c)** RT-qPCR of classic naïve (*Zfp42*) and primed (*Dnmt3b* and *Fgf5*) markers to assess the efficacy of conversion. Each grey line is an independent conversion, and mean tendency is represented by a black line (n=3). Source data are provided as Supplementary Data 10. **(d, e)** Heatmap showing the relative levels of enrichment (median $\log_2$ fold-change of ChIP-seq signal (RPKM+1) over input signal (RPKM+1)) for selected core and primed pluripotency transcription factors (OCT4, SOX2, OTX2 and ZIC2), enhancer marks (P300 and H3K27ac), and chromatin accessibility (median $\log_2$ fold-change of ATAC-seq signal (FPKM+1) over input signal (FPKM+1)) at PU, DM and INT subregions in EpiLCs (**d**) and EpiSCs (**e**). Enrichment scores for each feature are scaled by dividing by the mean enrichment score for that feature across all subregions. See Supplementary Data 1 for dataset accession numbers.

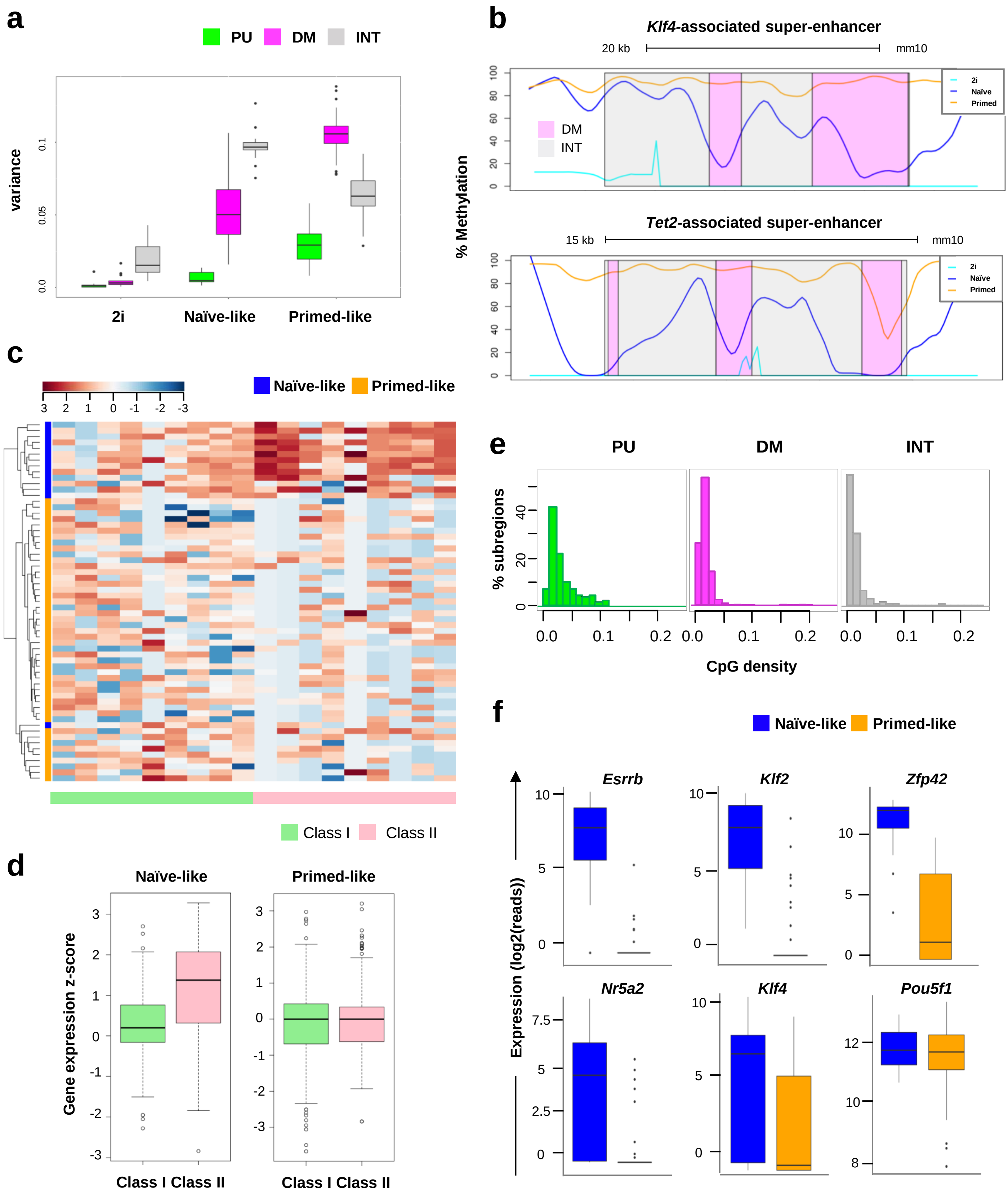

a
PU
DM
INT
variance
0.0
0.05
0.1
2i
Naïve-like
Primed-like
b
Klf4-associated super-enhancer
20 kb
mm10
% Methylation
DM
INT
2i
Naïve
Primed
Tet2-associated super-enhancer
15 kb
mm10
c
3 2 1 0 -1 -2 -3
Naïve-like
Primed-like
Class I
Class II
d
Naïve-like
Primed-like
Gene expression z-score
Class I Class II
Class I Class II
e
PU
DM
INT
% subregions
CpG density
f
Naïve-like
Primed-like
Expression (log2(reads))
Esrrb
Klf2
Zfp42
Nr5a2
Klf4
Pou5f1

Supplementary Figure 6. Related to Figure 4 - Differential CpG methylation dynamics at PU and DM super-enhancer subregions and impact on gene expression in ESCs

(**a**) Boxplots representing the distribution of methylation variances among PU (green), DM (magenta) and INT (grey) subregions in each individual 2i-ESC analysed, "naïve-like" and "primed-like" ser-ESC as shown in Fig. 4a. (**b**) Visualisation of CpG methylation profiles along the *Klf4* and *Tet2*-associated (Class II) SEs in "naïve-like" (dark blue) and "primed-like" (orange) single-cell clusters within ser-ESC population. 2i-ESC population (light blue) is included for reference. **(c)** Heatmap of gene expression z-score (relative to bulk ser-ESCs) of selected Class I (*Klf13*, *Lefty1*, *Med13l*, *Nanog*, *Otx2*, *Pou5f1*, *Smarcad1*, *Sox2* and *Tet1*) and Class II (*Esrrb*, *Klf2*, *Klf4*, *Klf5*, *Tbx3*, *Tdh*, *Tet2*, *Tfcp2l1*, and *Zfp42*) SE associated gene candidates in individual ser-ESCs. **(d)** Gene expression z-score (relative to bulk ser-ESCs) of Class I and Class II candidates in "naïve-like" and "primed-like" single-cell clusters within ser-ESC population as in (c). **(e)** Distribution of CpG density across PU, DM and INT subregions. CpG density was calculated as (CG dinucleotide count/length) for each subregion. **(f)** Expression (log2(reads)) of selected genes (*Esrrb, Klf2, Zfp42, Nr5a2, Klf4 and Pou5f1*) across "naïve-like" and "primed-like" single-cell clusters within ser-ESC population. See Supplementary Data 1 for dataset accession numbers.

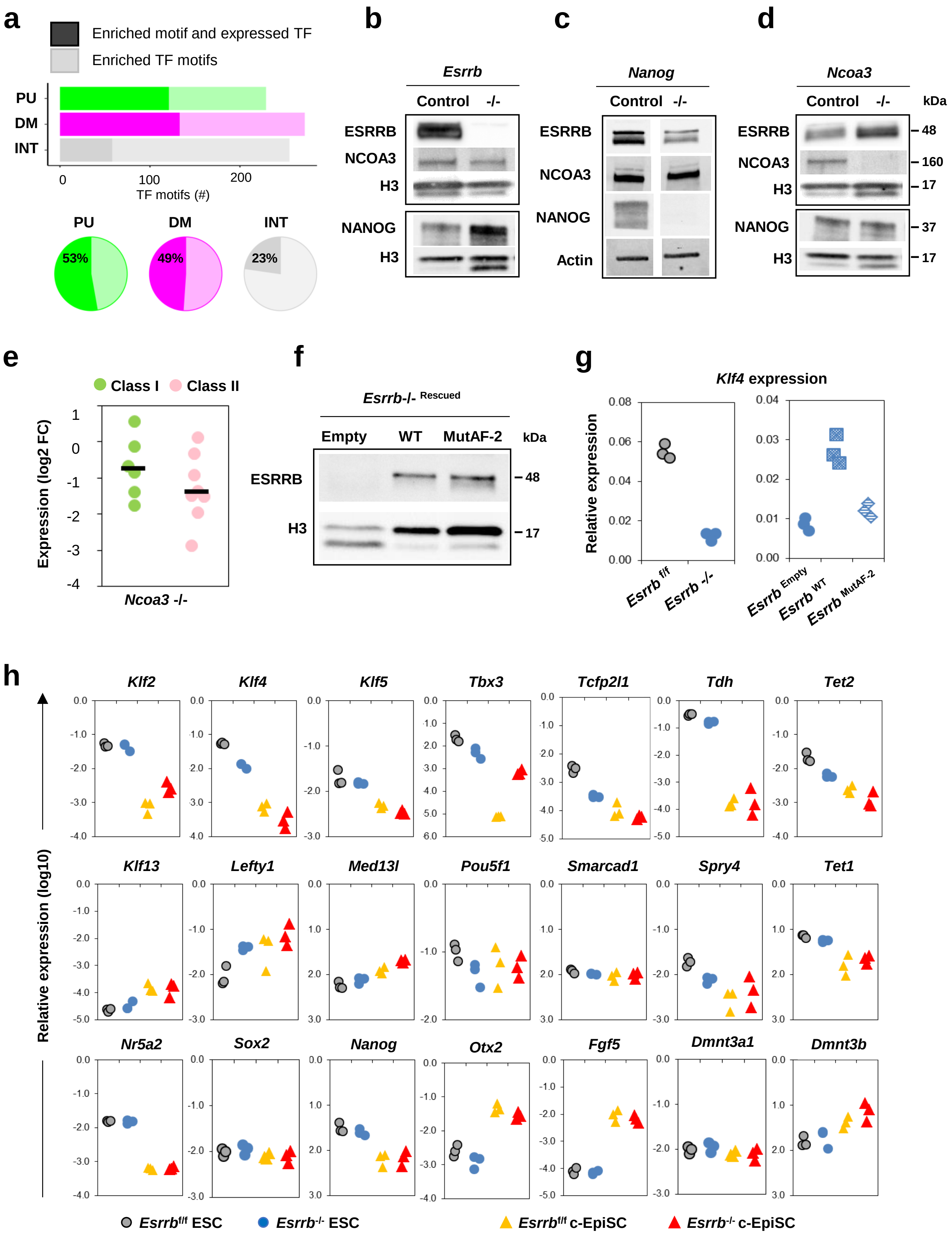
a
Enriched motif and expressed TF
Enriched TF motifs
PU
DM
INT
0
100
200
TF motifs (#)
53%
49%
23%
b
Esrrb
Control
-/-
ESRRB
NCOA3
H3
NANOG
H3
c
Nanog
Control
-/-
ESRRB
NCOA3
NANOG
Actin
d
Ncoa3
Control
-/-
kDa
ESRRB
NCOA3
H3
NANOG
H3
48
160
17
37
17
e
Class I
Class II
Expression (log2 FC)
Ncoa3 -/-
f
Esrrb-/- Rescued
Empty
WT
MutAF-2
kDa
ESRRB
H3
48
17
g
Klf4 expression
Relative expression
Esrrb f/f
Esrrb -/-
Esrrb Empty
Esrrb WT
Esrrb MutAF-2
h
Klf2
Klf4
Klf5
Tbx3
Tcfp2l1
Tdh
Tet2
Klf13
Lefty1
Med13l
Pou5f1
Smarcad1
Spry4
Tet1
Nr5a2
Sox2
Nanog
Otx2
Fgf5
Dmnt3a1
Dmnt3b
Relative expression (log10)
Esrrbf/f ESC
Esrrb-/- ESC
Esrrbf/f c-EpiSC
Esrrb-/- c-EpiSC

Supplementary Figure 7. Related to Figure 5 - Comparative gene expression analyses of Class I and Class II super-enhancer associated genes in ESCs depleted for ESRRB, NANOG or NCOA3

(a) Barplots depicting the number of statistically enriched motifs per SE subregion category (PU, green; DM, magenta; INT, grey) (padj < 0. 05, hypergeometric test). The dark area per bar displays the number of motifs for which at least one associated transcription factor is expressed in ESCs (RPKM ≥ 1). TF motifs enriched in INT subregion correspond to TFs significantly less often expressed than in other SE subregions (pie-chart: 23%; p-value < 3 x 10^{-6}, Fisher exact test). **(b, c, d)** Western blot characterization of ESCs constitutively depleted for *Esrrb* (b) *Nanog* (c), or *Ncoa3* (d) and their respective controls. A representative blot (from three technical replicates and 2 biological replicates) is shown. H3 or Actin serves as internal loading controls. **(e)** Expression changes (RT-qPCR) relative to matching control ESCs of selected class I (light green) and class II (pink) SE-associated genes in *Ncoa3*$^{-/-}$ ESCs: *Pou5f1* (*Oct4), Smarcad1, Otx2, Med13l, Nanog and Tet1* for Class I; and *Esrrb, Tfcp2l1, Klf4, Klf2, Klf5, Tdh, Tbx3* and *Tet2* for Class II. Medians are indicated by bars. The expression behaviour was not significantly different in one compared to the other class (Mann-Whitney test). **(f)** Western blot showing ESRRB expression in *Esrrb*$^{-/-}$ ESCs transfected with an empty vector, wild-type (WT) or mutant AF-2 (MutAF-2) form of *Esrrb*. **(g)** Expression level (RT-qPCR) of *Klf4* in *Esrrb*$^{f/f}$ and *Esrrb*$^{-/-}$ ESCs (left) and *Esrrb*$^{-/-}$ ESCs transfected with an empty vector, WT or MutAF-2 form of *Esrrb* (right panel). **(h)** Dot plots showing the expression levels obtained by RT-qPCR of selected genes in *Esrrb*$^{f/f}$, *Esrrb*$^{-/-}$ ESCs (grey and blue, respectively) and corresponding converted EpiSCs (c-EpiSCs)(orange and red, respectively). Expression data in (e, g and h) are representative of three independent experiments (n=3). Source data are provided as Supplementary Data 10.

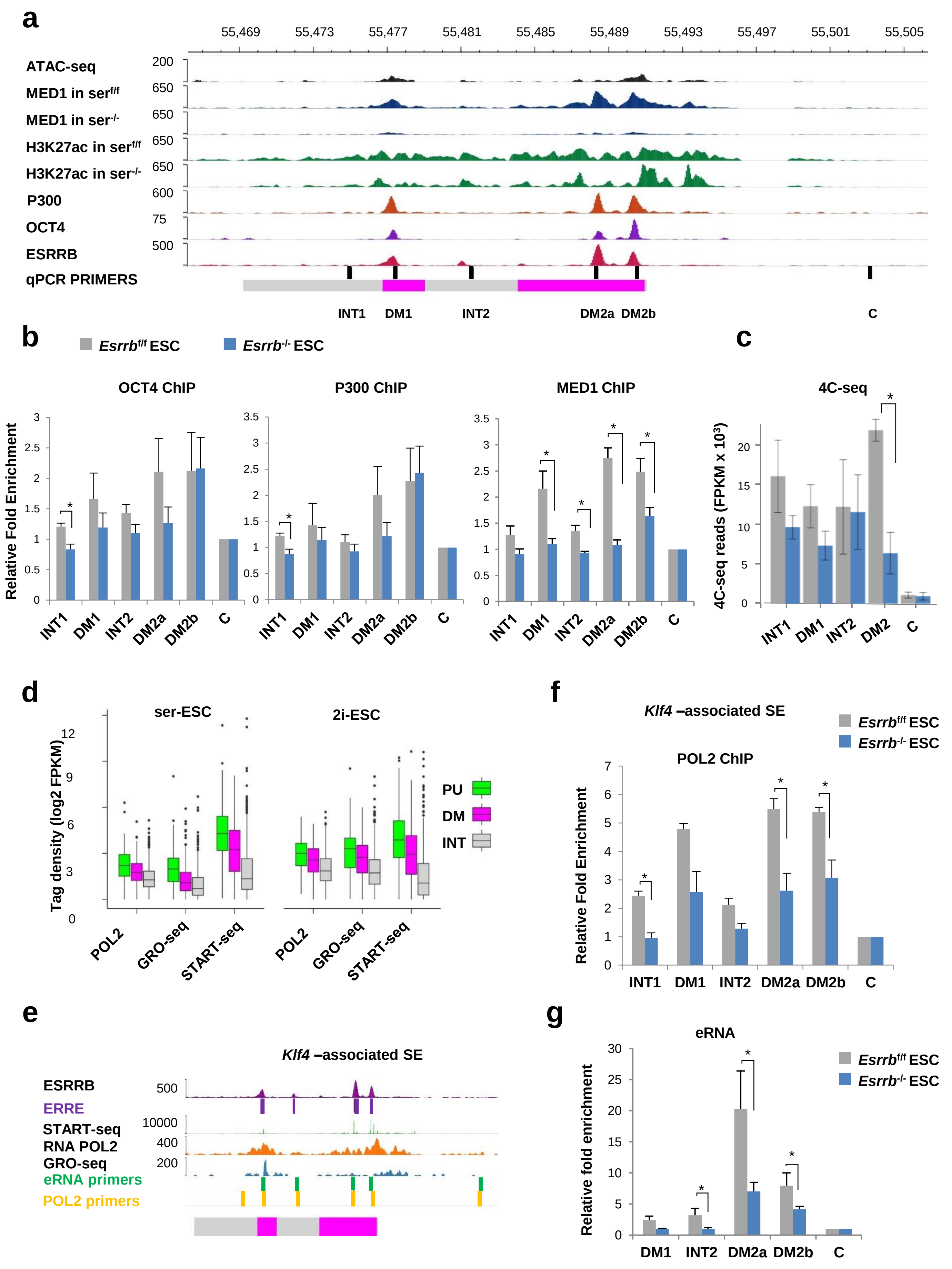

a
55,469 55,473 55,477 55,481 55,485 55,489 55,493 55,497 55,501 55,505
ATAC-seq 200
MED1 in ser f/f 650
MED1 in ser -/- 650
H3K27ac in ser f/f 650
H3K27ac in ser -/- 650
P300 600
OCT4 75
ESRRB 500
qPCR PRIMERS
INT1 DM1 INT2 DM2a DM2b C
b
Esrrb f/f ESC
Esrrb -/- ESC
OCT4 ChIP
P300 ChIP
MED1 ChIP
Relative Fold Enrichment
INT1 DM1 INT2 DM2a DM2b C
c
4C-seq
4C-seq reads (FPKM x 10³)
INT1 DM1 INT2 DM2 C
d
ser-ESC
2i-ESC
Tag density (log2 FPKM)
PU
DM
INT
POL2 GRO-seq START-seq
e
Klf4 –associated SE
ESRRB 500
ERRE
START-seq 10000
RNA POL2 400
GRO-seq 200
eRNA primers
POL2 primers
f
Klf4 –associated SE
POL2 ChIP
Esrrb f/f ESC
Esrrb -/- ESC
Relative Fold Enrichment
INT1 DM1 INT2 DM2a DM2b C
g
eRNA
Esrrb f/f ESC
Esrrb -/- ESC
Relative fold enrichment
DM1 INT2 DM2a DM2b C

Supplementary Figure 8. Related to Figure 6 – Analysis of protein binding profiles and eRNA transcription along the *Klf4*-associated super-enhancer upon ESRRB depletion in ESCs

(a) Detailed view of the *Klf4* SE locus showing ChIP-seq tracks of MED1 and H3K27ac in *Esrrb* $^{-/-}$ (ser$^{-/-}$)and control (ser$^{f/f}$) ESCs grown under serum/LIF conditions, as well as ATAC, P300, OCT4 and ESRRB in wild-type ESCs (serum/LIF). The localization of qPCR primers used for ChIP-qPCR and the name of amplified sites are also indicated along the *Klf4*-associated SE with DM (magenta) and INT (grey) subregions depicted. **(b)** ChIP-qPCR for OCT4, P300 and MED1 for all sites shown in (a) in *Esrrb*$^{-/-}$ (blue bars) and matching control ($^{f/f}$) (grey bars) ESCs grown under serum/LIF conditions. Data are expressed as fold enrichment over input and normalised to a flanking control (C) region. Data are represented by barplots of means ± s.e.m. of three or four independent experiments (n=3 for OCT4 and P300; n= 4 for MED1). Statistically significant difference (Mann-Whitney U test, * p-value <0.05). **(c)** 4C-seq reads at *Klf4*-associated SE subregions (INT1, DM1, INT2 and DM2) and a flanking control (C) region shown in (a) in *Esrrb*$^{-/-}$ and matching control ($^{f/f}$) ESCs grown under serum/LIF conditions. Data are represented by barplots of the average intensities ± s.e.m. of three independent experiments (n=3). Statistically significant difference (Two-sample T-test, * p-value =0.01). **(d)** Tag density of RNA polymerase II (POL2), GRO-seq and START-seq over PU (green), DM (magenta) and INT (grey) subregions in serum/LIF (ser) and 2i/LIF (2i) ESCs. **(e)** view of *Klf4* SE locus showing ChIP-seq tracks of ESRRB, START-seq, RNA POL2 and GRO-seq tracks, and positions of best ESRRB peak (ERRE), eRNA and POL2 qPCR primers. **(f)** ChIP-qPCR for POL2 at the sites shown in (e) along the *Klf4*-associated SE in *Esrrb*$^{-/-}$ and matching control ($^{f/f}$) ESCs grown under serum/LIF conditions. Data are expressed as fold enrichment over input and normalised to a flanking (C) region. Data are represented as barplots of means ± s.e.m. of independent experiments (n=4). Statistically significant difference (Mann-Whitney U test, * p-value <0.05). **(g)** Expression of eRNA at sites shown in (e) along the *Klf4*-associated SE as assessed by ChIP-qPCR in *Esrrb*$^{-/-}$ and matching control ($^{f/f}$) ESCs grown under serum/LIF conditions. Data are normalised to a flanking (C) region and represented as barplots of means ± s.e.m. of biological replicates (n= 3 for control ESCs; n=2 for *Esrrb*$^{-/-}$ ESCs). Statistically significant difference (Mann-Whitney U test, * p-value <0.05). See Supplementary Data 1 for dataset accession numbers. Source data are provided as Supplementary Data 10.

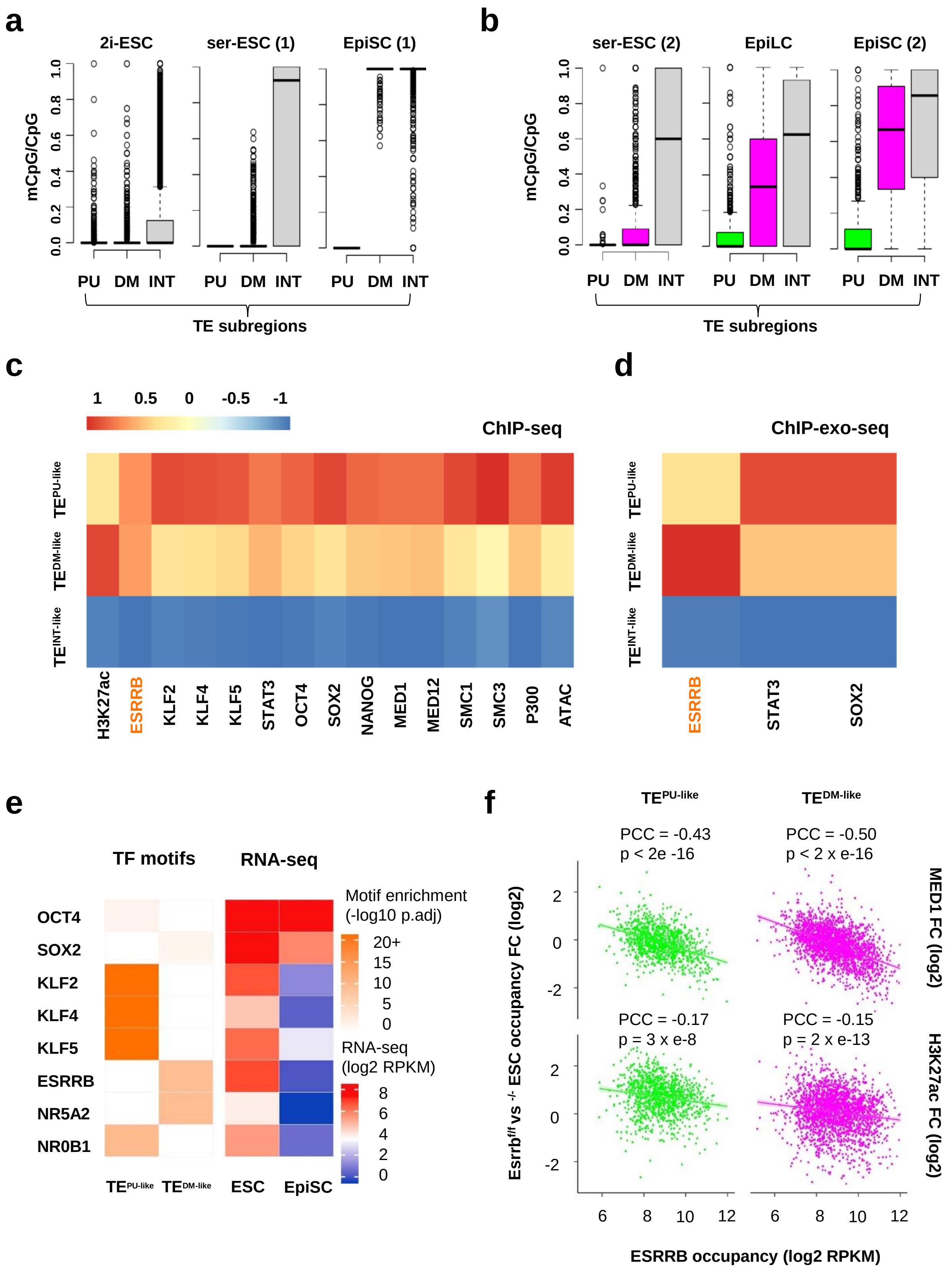
a
2i-ESC
ser-ESC (1)
EpiSC (1)
mCpG/CpG
PU DM INT
TE subregions
b
ser-ESC (2)
EpiLC
EpiSC (2)
mCpG/CpG
PU DM INT
TE subregions
c
ChIP-seq
TE PU-like
TE DM-like
TE INT-like
H3K27ac
ESRRB
KLF2
KLF4
KLF5
STAT3
OCT4
SOX2
NANOG
MED1
MED12
SMC1
SMC3
P300
ATAC
d
ChIP-exo-seq
ESRRB
STAT3
SOX2
e
TF motifs
RNA-seq
OCT4
SOX2
KLF2
KLF4
KLF5
ESRRB
NR5A2
NR0B1
TE PU-like
TE DM-like
ESC
EpiSC
Motif enrichment (-log10 p.adj)
RNA-seq (log2 RPKM)
f
TE PU-like
TE DM-like
PCC = -0.43 p < 2e -16
PCC = -0.50 p < 2 x e-16
PCC = -0.17 p = 3 x e-8
PCC = -0.15 p = 2 x e-13
Esrrb f/f vs -/- ESC occupancy FC (log2)
MED1 FC (log2)
H3K27ac FC (log2)
ESRRB occupancy (log2 RPKM)

Supplementary Figure 9 : Related to Figures 5 and 6 – Typical-enhancers (TE) partition into distinct enhancer networks in ESCs

(a) CpG methylation levels (mCpG/CpG) at PU-like, DM-like and INT-like TE subregions in 2i-ESCs and ser-ESCs (1), and EpiSCs (1). Theses cell lines are the same as in Supplementary Figure 4d. **(b)** CpG methylation levels (mCpG/CpG) at PU-like (green), DM-like (magenta) and INT-like (grey) TE subregions in an independent ser-ESC line (2), in EpiLCs and an independent EpiSC (2) line as in Fig. 2c. **(c)** Heatmap showing the relative levels of enrichment (median $\log_2$ fold-change of ChIP-seq signal (RPKM+1) over input signal (RPKM+1)) across PU-like, DM-like and INT-like TE subregions in ESCs (serum/LIF) for transcription factors, essential enhancer constituents and chromatin accessibility (ATAC-seq) as in Fig. 5c**.** **(d)** Heatmap showing the relative levels of enrichment (as in c) across PU-like, DM-like and INT-like TE subregions in ESCs (serum/LIF) for ESRRB, SOX2 and STAT3 in independent ChIP-EXO datasets as in Fig. 5d. In (c) and (d), enrichment scores for each feature are scaled by dividing by the mean enrichment score for that feature across all subregions. **(e)** TF motif enrichment in PU-like, DM-like and INT-like TE subregions, and gene expression of corresponding TFs in ESCs (serum/LIF) and EpiSCs. **(f)** MED1 (top) and H3K27ac (bottom) occupancy changes in *Esrrb*$^{-/-}$ compared to control ($^{f/f}$) ESCs grown under serum/LIF conditions. PCC: Pearson's correlation coefficient. Publicly available datasets used here are listed in Supplementary Data 1.

Supplementary references

1. Habibi, E. et al. Whole-Genome Bisulfite Sequencing of Two Distinct Interconvertible DNA Methylomes of Mouse Embryonic Stem Cells. Cell Stem Cell **13**, 360–369 (2013).
2. Duncan, C. G. et al. Base-Resolution Analysis of DNA Methylation Patterns Downstream of Dnmt3a in Mouse Naïve B Cells. G3 (Bethesda) **8**, 805–813 (2018).
3. Joshi, O. et al. Dynamic Reorganization of Extremely Long-Range Promoter-Promoter Interactions between Two States of Pluripotency. Cell Stem Cell **17**, 748–757 (2015).
4. Sahlén, P. et al. Genome-wide mapping of promoter-anchored interactions with close to single-enhancer resolution. Genome Biol. **16**, 156 (2015).
5. Novo, C. L. et al. The pluripotency factor Nanog regulates pericentromeric heterochromatin organization in mouse embryonic stem cells. Genes Dev. **30**, 1101-1115 (2016).
6. Dowen, J. M. et al. Control of Cell Identity Genes Occurs in Insulated Neighborhoods in Mammalian Chromosomes. Cell **159**, 374–387 (2014).
7. Stadler, M. B. et al. DNA-binding factors shape the mouse methylome at distal regulatory regions. Nature **480**, 490–495 (2011).
8. Zylicz, J. J. et al. Chromatin dynamics and the role of G9a in gene regulation and enhancer silencing during early mouse development. eLife **4**, e09571 (2015).
9. Marks, H. et al. The Transcriptional and Epigenomic Foundations of Ground State Pluripotency. Cell **149**, 590–604 (2012).
10. Veillard, A. C. et al. Stable methylation at promoters distinguishes epiblast stem cells from embryonic stem cells and the in vivo epiblasts. Stem Cells and Development **23**, 2014–29 (2014).
11. Cabezas-Wallscheid, N. et al. Identification of regulatory networks in HSCs and their immediate progeny via integrated proteome, transcriptome, and DNA methylome analysis. Cell Stem Cell **15**, 507–522 (2014).
