# Supplementary material for: Dynamic CpG methylation delineates subregions within super-enhancers selectively decommissioned at the exit from naive pluripotency
Source: Nat Commun. 2020 Feb 28;11:1112. doi: 10.1038/s41467-020-14916-7 (PMC7048827; doi:10.1038/s41467-020-14916-7)
Supplement: Supplementary file 3 — Description of Additional Supplementary Files [file 41467_2020_14916_MOESM3_ESM.pdf]

Description of Additional Supplementary Files

File Name: Supplementary Data 1

Description: List of all datasets used with accession numbers

File Name: Supplementary Data 2

Description: Capture Hi-C data (Joshi et al. and Sahlen et al.)

File Name: Supplementary Data 3

Description: CpG methylation and ATAC-seq at SE subregions

File Name: Supplementary Data 4

Description: ROC analysis in naïve-like and primed-like single ESC clusters

File Name: Supplementary Data 5

Description: Motif enrichment and expression of corresponding transcription factors (TFs) at SE subregions

File Name: Supplementary Data 6

Description: List of qPCR primers used for expression analysis as well as 5mC and ChIP assays

File Name: Supplementary Data 7

Description: 4C-seq primers and mapping statistics

File Name: Supplementary Data 8

Description: Coefficients and statistics for logistic regression models relating epigenetic features to SE subregion status

File Name: Supplementary Data 9

Description: Coefficients and statistics for multivariable linear regression model relating ESRRB and OCT4 DNA-binding at PU vs DM to the loss of MED1 binding upon constitutive depletion of ESRRB

File Name: Supplementary Data 10

Description: Source Data file for all relevant Main Figures and Supplementary Figures
