# Supplementary material for: Dynamic CpG methylation delineates subregions within super-enhancers selectively decommissioned at the exit from naive pluripotency
Source: Nat Commun. 2020 Feb 28;11:1112. doi: 10.1038/s41467-020-14916-7 (PMC7048827; doi:10.1038/s41467-020-14916-7)
Supplement: Supplementary file 14 — Reporting Summary [file 41467_2020_14916_MOESM14_ESM.pdf]

nature research

Corresponding author(s): V. Azuara and A. Jouneau

Last updated by author(s): Jan 20, 2020

Reporting Summary

Nature Research wishes to improve the reproducibility of the work that we publish. This form provides structure for consistency and transparency in reporting. For further information on Nature Research policies, see Authors & Referees and the Editorial Policy Checklist.

Statistics

For all statistical analyses, confirm that the following items are present in the figure legend, table legend, main text, or Methods section.

n/a	Confirmed	
☐	☒	The exact sample size (*n*) for each experimental group/condition, given as a discrete number and unit of measurement
☐	☒	A statement on whether measurements were taken from distinct samples or whether the same sample was measured repeatedly
☐	☒	The statistical test(s) used AND whether they are one- or two-sided<br>*Only common tests should be described solely by name; describe more complex techniques in the Methods section.*
☒	☐	A description of all covariates tested
☐	☒	A description of any assumptions or corrections, such as tests of normality and adjustment for multiple comparisons
☒	☐	A full description of the statistical parameters including central tendency (e.g. means) or other basic estimates (e.g. regression coefficient) AND variation (e.g. standard deviation) or associated estimates of uncertainty (e.g. confidence intervals)
☐	☒	For null hypothesis testing, the test statistic (e.g. *F*, *t*, *r*) with confidence intervals, effect sizes, degrees of freedom and *P* value noted<br>*Give P values as exact values whenever suitable.*
☒	☐	For Bayesian analysis, information on the choice of priors and Markov chain Monte Carlo settings
☒	☐	For hierarchical and complex designs, identification of the appropriate level for tests and full reporting of outcomes
☐	☒	Estimates of effect sizes (e.g. Cohen's *d*, Pearson's *r*), indicating how they were calculated

Our web collection on statistics for biologists contains articles on many of the points above.

Software and code

Policy information about availability of computer code

Data collection	Real-time pPCR: StepOne plus Applied Biosystem<br>Western blot: Chemidoc Touch imaging system (Biorad) and ImageJ<br>Sequencing: Illumina Genome Analyzer II (ChIP-seq and 4C) andIllumina HiSeq200 (BS-seq)<br>SRAtoolkit v2.8.2 (prefetch and fastq-dump) was used to obtain sequencing data from GEO and extract fastq files for analysis.
Data analysis	Sequencing data analysis involved: Trimmomatic v0.33, Bowtie2 v2.2.3, Picard Tools v1.128, BedTools v2.22.1.<br>R v3.5.1 was used in sequencing data analysis, with the following packages: plotCoverage, GenomicRanges v1.30.3, IRanges 2.12.0, NMF v0.21.0, ggplot2 v3.0.0, BSgenome, FourCSeq, data.table.<br>Mmusculus.UCSC.mm9 v1.4.0, IGV, Washu epigenome browser.<br>BS methylation data was analysed using tileHMM R package<br>Github repository for codes used throughout the figures: ihttps://github.com/edcurry/esc-se-regions

For manuscripts utilizing custom algorithms or software that are central to the research but not yet described in published literature, software must be made available to editors/reviewers. We strongly encourage code deposition in a community repository (e.g. GitHub). See the Nature Research guidelines for submitting code & software for further information.

Data

Policy information about availability of data

All manuscripts must include a data availability statement. This statement should provide the following information, where applicable:

- Accession codes, unique identifiers, or web links for publicly available datasets
- A list of figures that have associated raw data
- A description of any restrictions on data availability

BS-seq, ChIP-seq and 4C-seq data that support the findings of this study have been deposited in the GEO database under accession numbers GSE124476 and GSE129179.

Accession numbers of publicly available datasets used in this study are provided in Supplementary Table 1.

Field-specific reporting

Please select the one below that is the best fit for your research. If you are not sure, read the appropriate sections before making your selection.

☒ Life sciences ☐ Behavioural & social sciences ☐ Ecological, evolutionary & environmental sciences

For a reference copy of the document with all sections, see nature.com/documents/nr-reporting-summary-flat.pdf

Life sciences study design

All studies must disclose on these points even when the disclosure is negative.

Sample size	No statistical methods were used to predetermine sample size.
Data exclusions	No data were excluded from the analyses.
Replication	All attempts at data replication were successful.
Randomization	No randomization methods were used in this study.
Blinding	Investigators were not blinded; all findings are supported by quantitative measurements.

Behavioural & social sciences study design

All studies must disclose on these points even when the disclosure is negative.

Study description	*Briefly describe the study type including whether data are quantitative, qualitative, or mixed-methods (e.g. qualitative cross-sectional, quantitative experimental, mixed-methods case study).*
Research sample	*State the research sample (e.g. Harvard university undergraduates, villagers in rural India) and provide relevant demographic information (e.g. age, sex) and indicate whether the sample is representative. Provide a rationale for the study sample chosen. For studies involving existing datasets, please describe the dataset and source.*
Sampling strategy	*Describe the sampling procedure (e.g. random, snowball, stratified, convenience). Describe the statistical methods that were used to predetermine sample size OR if no sample-size calculation was performed, describe how sample sizes were chosen and provide a rationale for why these sample sizes are sufficient. For qualitative data, please indicate whether data saturation was considered, and what criteria were used to decide that no further sampling was needed.*
Data collection	*Provide details about the data collection procedure, including the instruments or devices used to record the data (e.g. pen and paper, computer, eye tracker, video or audio equipment) whether anyone was present besides the participant(s) and the researcher, and whether the researcher was blind to experimental condition and/or the study hypothesis during data collection.*
Timing	*Indicate the start and stop dates of data collection. If there is a gap between collection periods, state the dates for each sample cohort.*
Data exclusions	*If no data were excluded from the analyses, state so OR if data were excluded, provide the exact number of exclusions and the rationale behind them, indicating whether exclusion criteria were pre-established.*
Non-participation	*State how many participants dropped out/declined participation and the reason(s) given OR provide response rate OR state that no participants dropped out/declined participation.*
Randomization	*If participants were not allocated into experimental groups, state so OR describe how participants were allocated to groups, and if allocation was not random, describe how covariates were controlled.*

Ecological, evolutionary & environmental sciences study design

All studies must disclose on these points even when the disclosure is negative.

Study description	*Briefly describe the study. For quantitative data include treatment factors and interactions, design structure (e.g. factorial, nested, hierarchical), nature and number of experimental units and replicates.*
Research sample	*Describe the research sample (e.g. a group of tagged Passer domesticus, all Stenocereus thurberi within Organ Pipe Cactus National Monument), and provide a rationale for the sample choice. When relevant, describe the organism taxa, source, sex, age range and*

	any manipulations. State what population the sample is meant to represent when applicable. For studies involving existing datasets, describe the data and its source.
Sampling strategy	*Note the sampling procedure. Describe the statistical methods that were used to predetermine sample size OR if no sample-size calculation was performed, describe how sample sizes were chosen and provide a rationale for why these sample sizes are sufficient.*
Data collection	*Describe the data collection procedure, including who recorded the data and how.*
Timing and spatial scale	*Indicate the start and stop dates of data collection, noting the frequency and periodicity of sampling and providing a rationale for these choices. If there is a gap between collection periods, state the dates for each sample cohort. Specify the spatial scale from which the data are taken*
Data exclusions	*If no data were excluded from the analyses, state so OR if data were excluded, describe the exclusions and the rationale behind them, indicating whether exclusion criteria were pre-established.*
Reproducibility	*Describe the measures taken to verify the reproducibility of experimental findings. For each experiment, note whether any attempts to repeat the experiment failed OR state that all attempts to repeat the experiment were successful.*
Randomization	*Describe how samples/organisms/participants were allocated into groups. If allocation was not random, describe how covariates were controlled. If this is not relevant to your study, explain why.*
Blinding	*Describe the extent of blinding used during data acquisition and analysis. If blinding was not possible, describe why OR explain why blinding was not relevant to your study.*

Did the study involve field work? ☐ Yes ☐ No

Field work, collection and transport

Field conditions	*Describe the study conditions for field work, providing relevant parameters (e.g. temperature, rainfall).*
Location	*State the location of the sampling or experiment, providing relevant parameters (e.g. latitude and longitude, elevation, water depth).*
Access and import/export	*Describe the efforts you have made to access habitats and to collect and import/export your samples in a responsible manner and in compliance with local, national and international laws, noting any permits that were obtained (give the name of the issuing authority, the date of issue, and any identifying information).*
Disturbance	*Describe any disturbance caused by the study and how it was minimized.*

Reporting for specific materials, systems and methods

We require information from authors about some types of materials, experimental systems and methods used in many studies. Here, indicate whether each material, system or method listed is relevant to your study. If you are not sure if a list item applies to your research, read the appropriate section before selecting a response.

Materials & experimental systems

n/a	Involved in the study	
☐	☒	Antibodies
☐	☒	Eukaryotic cell lines
☒	☐	Palaeontology
☒	☐	Animals and other organisms
☒	☐	Human research participants
☒	☐	Clinical data

Methods

n/a	Involved in the study	
☐	☒	ChIP-seq
☒	☐	Flow cytometry
☒	☐	MRI-based neuroimaging

Antibodies

Antibodies used	ESRRB (R&D H6705)<br>NCOA3 (Santa Cruz Sc9119)<br>NANOG (Abcam, 80892)<br>ACTIN (Sigma, A5441)<br>H3 (Abcam ab1791)<br>MED1 (A300-793A Bethyl Laboratories)<br>OCT4 (sc-8628 SantaCruz),<br>p300 (sc-585 SantaCruz)<br>POL2 (Clone 8WG16 MMS-126R, Covance).<br>H3K27ac (Ab4729 Abcam)

Validation	Antibodies were chosen based on previous literature. Validation and quality control is available from the manufacturers using the catalog number of each antibody.

Eukaryotic cell lines

Policy information about cell lines

Cell line source(s)	Mouse wild-type R1 embryonic stem cells were purchased from ATCC.<br>Mouse ESRRB-/- embryonic stem cells (ESCs) and matching control cells were kindly provided by Austin Smith - see reference Martello, G. et al. Esrrb is a pivotal target of the Gsk3/Tcf3 axis regulating embryonic stem cell self-renewal. Cell Stem Cell 11, 491–504 (2012).<br>Mouse NANOG -/- and control ESCs were kindly provided by Ian Chambers - see reference Chambers, I. et al. Nanog safeguards pluripotency and mediates germline development. Nature 450, 1230–1234 (2007).<br>Mouse NCOA3-/- and control ESCs were derived from mutant and wild-type C57/129 mice and kindly provided by Austin Cooney.<br>Mouse epiblast stem cells (EpiSCs; 8.73) were derived from B6D2 mice (epiblast E6.5) by Alice Jouneau - see reference Veillard, A. C. et al. Stable methylation at promoters distinguishes epiblast stem cells from embryonic stem cells and the in vivo epiblasts. Stem Cells and Development 23, 2014–29 (2014).
Authentication	These cells were authenticated based on different culture condition requirements and phenotypic validations (e.g. RT-qPCR, western blot).
Mycoplasma contamination	Testing for mycoplasma contamination was routinely performed using MycoZap assay procedure (Lonza).
Commonly misidentified lines<br>(See ICLAC register)	*Name any commonly misidentified cell lines used in the study and provide a rationale for their use.*

Palaeontology

Specimen provenance	*Provide provenance information for specimens and describe permits that were obtained for the work (including the name of the issuing authority, the date of issue, and any identifying information).*
Specimen deposition	*Indicate where the specimens have been deposited to permit free access by other researchers.*
Dating methods	*If new dates are provided, describe how they were obtained (e.g. collection, storage, sample pretreatment and measurement), where they were obtained (i.e. lab name), the calibration program and the protocol for quality assurance OR state that no new dates are provided.*

☐ Tick this box to confirm that the raw and calibrated dates are available in the paper or in Supplementary Information.

Animals and other organisms

Policy information about studies involving animals; ARRIVE guidelines recommended for reporting animal research

Laboratory animals	*For laboratory animals, report species, strain, sex and age OR state that the study did not involve laboratory animals.*
Wild animals	*Provide details on animals observed in or captured in the field; report species, sex and age where possible. Describe how animals were caught and transported and what happened to captive animals after the study (if killed, explain why and describe method; if released, say where and when) OR state that the study did not involve wild animals.*
Field-collected samples	*For laboratory work with field-collected samples, describe all relevant parameters such as housing, maintenance, temperature, photoperiod and end-of-experiment protocol OR state that the study did not involve samples collected from the field.*
Ethics oversight	*Identify the organization(s) that approved or provided guidance on the study protocol, OR state that no ethical approval or guidance was required and explain why not.*

Note that full information on the approval of the study protocol must also be provided in the manuscript.

Human research participants

Policy information about studies involving human research participants

Population characteristics	*Describe the covariate-relevant population characteristics of the human research participants (e.g. age, gender, genotypic information, past and current diagnosis and treatment categories). If you filled out the behavioural & social sciences study design questions and have nothing to add here, write "See above."*
Recruitment	*Describe how participants were recruited. Outline any potential self-selection bias or other biases that may be present and how these are likely to impact results.*
Ethics oversight	*Identify the organization(s) that approved the study protocol.*

Note that full information on the approval of the study protocol must also be provided in the manuscript.

Clinical data

Policy information about clinical studies

All manuscripts should comply with the ICMJE guidelines for publication of clinical research and a completed CONSORT checklist must be included with all submissions.

Clinical trial registration — *Provide the trial registration number from ClinicalTrials.gov or an equivalent agency.*

Study protocol — *Note where the full trial protocol can be accessed OR if not available, explain why.*

Data collection — *Describe the settings and locales of data collection, noting the time periods of recruitment and data collection.*

Outcomes — *Describe how you pre-defined primary and secondary outcome measures and how you assessed these measures.*

ChIP-seq

Data deposition

☒ Confirm that both raw and final processed data have been deposited in a public database such as GEO.

☐ Confirm that you have deposited or provided access to graph files (e.g. BED files) for the called peaks.

Data access links
May remain private before publication.

https://www.ncbi.nlm.nih.gov/geo/query/acc.cgi?acc=GSE139189

Files in database submission

GSE129179 Enhancer profiling in Esrrb-/- ES cells [ChIP-seq]
GSM3701992 InputDNA_Esrrb_wt_rep1 BED
GSM3701993 Med1_ChIP_Esrrb_wt_rep1 BED
GSM3701994 H3K27ac_ChIP_Esrrb_wt_rep1 BED
GSM3701995 H3K27ac_ChIP_Esrrb_wt_rep2 BED
GSM3701996 InputDNA_Esrrb_wt_rep2 BED
GSM3701997 Med1_ChIP_Esrrb_wt_rep2 BED
GSM3701998 InputDNA_Esrrb_KO_rep1 BED
GSM3701999 Med1_ChIP_Esrrb_KO_rep1 BED
GSM3702000 H3K27ac_ChIP_Esrrb_KO_rep1 BED
GSM3702001 H3K27ac_ChIP_Esrrb_KO_rep2 BED
GSM3702002 InputDNA_Esrrb_KO_rep2 BED
GSM3702003 Med1_ChIP_Esrrb_KO_rep2 BED

GSE139187 Enhancer profiling in Esrrb-/- ES cells [4C-seq]
GSM4133256 Esrrb_wt_serum_rep1 BEDGRAPH
GSM4133257 Esrrb_wt_serum_rep2 BEDGRAPH
GSM4133258 Esrrb_wt_serum_rep3 BEDGRAPH
GSM4133259 Esrrb_wt_2i_rep1 BEDGRAPH
GSM4133260 Esrrb_wt_2i_rep2 BEDGRAPH
GSM4133261 Esrrb_wt_2i_rep3 BEDGRAPH
GSM4133262 Esrrb_KO_serum_rep1 BEDGRAPH
GSM4133263 Esrrb_KO_serum_rep2 BEDGRAPH
GSM4133264 Esrrb_KO_serum_rep3 BEDGRAPH

Genome browser session (e.g. UCSC) — *Provide a link to an anonymized genome browser session for "Initial submission" and "Revised version" documents only, to enable peer review. Write "no longer applicable" for "Final submission" documents.*

Methodology

Replicates — 2 replicate libraries were produced for each ChIP antibody (Med1, H3K27ac, input), for each condition (Esrrb fl/fl, Esrrb -/-)

Sequencing depth

All libraries were sequenced with paired-end, 43-bp reads. Approximately 35 million reads were sequenced for each sample.
Table of sequencing depth and unique reads:
Sample NumberReadPairs NumberMapped PercentMapped
1-Esrrb-FlFl-input-1 49948090 48754711 97.61076149
2-Esrrb-FlFl-Med1 62809560 61843975 98.46267829
3-Esrrb-FlFl-H3K27ac 42504182 40500069 95.28490397
4-Esrrb-FlFl-H3K27ac 69738132 66270380 95.02746647
5-Esrrb-FlFl-input-2 68143752 65508823 96.13327866
6-Esrrb-FlFl-Med1 59467244 58578523 98.50552852
7-Esrrb-/-input-1 69467146 68475655 98.57271954
8-Esrrb-/-Med1 51694262 50239205 97.185264
9-Esrrb-/-H3K27ac 66327668 63124605 95.17084937

10-Esrrb-/-H3K27ac 51504504 50247823 97.56005611
11-Esrrb-/-input-2 75602466 73119660 96.7159722
12-Esrrb-/-Med1 62226800 61301788 98.51348294

Antibodies: MED1 : A300-793A Bethyl Laboratories
H3K27ac (Ab4729 Abcam)

Peak calling parameters: Peak calling wasn't performed for this study.

Data quality: Sequencing adapters were removed, low-quality reads were trimmed and filtered. Duplicate reads were removed, and reads mapping to ENCODE blacklisted regions were removed. Peak-calling wasn't performed for this study.

Software: Trimmomatic was used for adapter removal and read filtering. Picard Tools MarkDuplicates were used to remove duplicate reads. Bedtools intersect was used to remove reads mapping to ENCODE blacklisted regions. The R function countOverlaps (from package GenomicRanges) was used to count reads mapping to each SE subregion.

Flow Cytometry

Plots

Confirm that:

☐ The axis labels state the marker and fluorochrome used (e.g. CD4-FITC).

☐ The axis scales are clearly visible. Include numbers along axes only for bottom left plot of group (a 'group' is an analysis of identical markers).

☐ All plots are contour plots with outliers or pseudocolor plots.

☐ A numerical value for number of cells or percentage (with statistics) is provided.

Methodology

Sample preparation: *Describe the sample preparation, detailing the biological source of the cells and any tissue processing steps used.*

Instrument: *Identify the instrument used for data collection, specifying make and model number.*

Software: *Describe the software used to collect and analyze the flow cytometry data. For custom code that has been deposited into a community repository, provide accession details.*

Cell population abundance: *Describe the abundance of the relevant cell populations within post-sort fractions, providing details on the purity of the samples and how it was determined.*

Gating strategy: *Describe the gating strategy used for all relevant experiments, specifying the preliminary FSC/SSC gates of the starting cell population, indicating where boundaries between "positive" and "negative" staining cell populations are defined.*

☐ Tick this box to confirm that a figure exemplifying the gating strategy is provided in the Supplementary Information.

Magnetic resonance imaging

Experimental design

Design type: *Indicate task or resting state; event-related or block design.*

Design specifications: *Specify the number of blocks, trials or experimental units per session and/or subject, and specify the length of each trial or block (if trials are blocked) and interval between trials.*

Behavioral performance measures: *State number and/or type of variables recorded (e.g. correct button press, response time) and what statistics were used to establish that the subjects were performing the task as expected (e.g. mean, range, and/or standard deviation across subjects).*

Acquisition

Imaging type(s): *Specify: functional, structural, diffusion, perfusion.*

Field strength: *Specify in Tesla*

Sequence & imaging parameters: *Specify the pulse sequence type (gradient echo, spin echo, etc.), imaging type (EPI, spiral, etc.), field of view, matrix size, slice thickness, orientation and TE/TR/flip angle.*

Area of acquisition: *State whether a whole brain scan was used OR define the area of acquisition, describing how the region was determined.*

Diffusion MRI ☐ Used ☐ Not used

Preprocessing

Preprocessing software	*Provide detail on software version and revision number and on specific parameters (model/functions, brain extraction, segmentation, smoothing kernel size, etc.).*
Normalization	*If data were normalized/standardized, describe the approach(es): specify linear or non-linear and define image types used for transformation OR indicate that data were not normalized and explain rationale for lack of normalization.*
Normalization template	*Describe the template used for normalization/transformation, specifying subject space or group standardized space (e.g. original Talairach, MNI305, ICBM152) OR indicate that the data were not normalized.*
Noise and artifact removal	*Describe your procedure(s) for artifact and structured noise removal, specifying motion parameters, tissue signals and physiological signals (heart rate, respiration).*
Volume censoring	*Define your software and/or method and criteria for volume censoring, and state the extent of such censoring.*

Statistical modeling & inference

Model type and settings	*Specify type (mass univariate, multivariate, RSA, predictive, etc.) and describe essential details of the model at the first and second levels (e.g. fixed, random or mixed effects; drift or auto-correlation).*
Effect(s) tested	*Define precise effect in terms of the task or stimulus conditions instead of psychological concepts and indicate whether ANOVA or factorial designs were used.*

Specify type of analysis: ☐ Whole brain ☐ ROI-based ☐ Both

Statistic type for inference<br>(See Eklund et al. 2016)	*Specify voxel-wise or cluster-wise and report all relevant parameters for cluster-wise methods.*
Correction	*Describe the type of correction and how it is obtained for multiple comparisons (e.g. FWE, FDR, permutation or Monte Carlo).*

Models & analysis

n/a	Involved in the study
☐	☐ Functional and/or effective connectivity
☐	☐ Graph analysis
☐	☐ Multivariate modeling or predictive analysis

Functional and/or effective connectivity	*Report the measures of dependence used and the model details (e.g. Pearson correlation, partial correlation, mutual information).*
Graph analysis	*Report the dependent variable and connectivity measure, specifying weighted graph or binarized graph, subject- or group-level, and the global and/or node summaries used (e.g. clustering coefficient, efficiency, etc.).*
Multivariate modeling and predictive analysis	*Specify independent variables, features extraction and dimension reduction, model, training and evaluation metrics.*
